# On the scattering directionality of a dielectric particle dimer of High Refractive Index

**DOI:** 10.1038/s41598-018-26359-8

**Published:** 2018-05-22

**Authors:** Ángela I. Barreda, Hassan Saleh, Amélie Litman, Francisco González, Jean-Michel Geffrin, Fernando Moreno

**Affiliations:** 1 Group of Optics, Department of Applied Physics, University of Cantabria, Cantabria, 39005 Spain; 2 Aix-Marseille Univ, CNRS, Centrale Marseille, Institut Fresnel, Marseille, France; 3 Centre Commun de Ressources en Microondes CCRM, 5 rue Enrico Fermi, Marseille, 13453 France

## Abstract

Low-losses and directionality effects exhibited by High Refractive Index Dielectric particles make them attractive for applications where radiation direction control is relevant. For instance, isolated metallo-dielectric core-shell particles or aggregates (dimers) of High Refractive Index Dielectric particles have been proposed for building operational switching devices. Also, the possibility of using isolated High Refractive Index Dielectric particles for optimizing solar cells performance has been explored. Here, we present experimental evidence in the microwave range, that a High Refractive Index Dielectric dimer of spherical particles is more efficient for redirecting the incident radiation in the forward direction than the isolated case. In fact, we report two spectral regions in the dipolar spectral range where the incident intensity is mostly scattered in the forward direction. They correspond to the Zero-Backward condition (also observed for isolated particles) and to a new condition, denoted as “near Zero-Backward” condition, which comes from the interaction effects between the particles. The proposed configuration has implications in solar energy harvesting devices and in radiation guiding.

## Introduction

Solar energy constitutes one of the most important renewable energy sources. Its clean and non-polluting energy can be converted into electricity by photovoltaic devices like solar cells, which have become a powerful alternative for solving the problem of climate change. However, the high manufacturing costs due to thicknesses of crystalline silicon wafer (typically 200–300 µm), which they are made of, make them not fully competitive with the actual fossil fuel energy resources. For decreasing expenses, thin-film solar cells, whose thickness is about 1–2 µm, have been proposed^1^. Nevertheless, one of their main disadvantages is the low absorbance of the incident radiation.

In order to increase their efficiency, the use of subwavelength metallic particles on top of the photosensitive surface has been suggested^2,3^. Indeed, when incident light illuminates a small metallic particle, free electrons start to oscillate to the same frequency as the incident radiation. These coherent oscillations of the electron plasma depend on the material properties, the particle size and shape, and on the wavelength of the incoming radiation and result in particular surface charge distributions^4^. At the resonant frequencies, enhancements of the electric field can be observed in the particle surroundings^5^. This phenomenon has been exploited in many different applications like surface enhanced Raman spectroscopy (SERS), or photovoltaic devices among others^1–3,6–11^. Therefore, this kind of nanostructures can enhance the absorption of the incident radiation by means of two different mechanisms^1^. On one hand, metallic particles can help to couple the incident radiation into the substrate leading to an increment of the absorbed radiation. This is mainly due to the angular spread acquired by the scattered light in the dielectric, so the optical path length of the radiation in the photosensitive volume increases^1^. On the other hand, strong enhancements of the electric field in the particle surroundings stimulate the absorption of the incident radiation in the semiconductor wafer^1^. However, in spite of the good response of metallic particles in infrared (IR) and visible (VIS) spectral regions^12^, due to the Joule’s effect and consequently, to their inherent ohmic losses, the major part of the incident radiation is converted into heating. These photons do not generate electron-hole pairs and, in consequence, do not contribute to increase the electric current. This fact limits the utility of metallic particles in energy harvesting applications.

High Refractive Index Dielectric (HRID) particles seem to be a promising alternative to address this issue due to their low losses^13–23^. In addition, HRID particles can be designed to control the direction of the scattered radiation. Their interesting directionality properties arise from the coherence effects between the electric and magnetic resonances observed in the spectra^14^. As the absorption radiation is lower in NIR than in VIS spectral region^1^, in order to improve solar cells efficiency, directionality properties should be fulfilled in that spectral region. For that reason, in this work, we focus on the spectral range where the dipolar approximation holds (i.e. the scattered intensity is mainly described by means of the dipolar electric and magnetic scattering Mie coefficients, a_1_ and b_1_ respectively, rendering the other higher orders negligible). It was demonstrated that the directionality effects could be enhanced through the interference of dipolar and multipolar orders^24–28^. However, the directionality conditions originated by electric and magnetic dipoles are observed at longer wavelengths, which are the ones of interest here, than those involving multipolar resonances^25^.

In particular, the scattered radiation obtained from a single HRID spherical particle can be concentrated either in the backscattering region or in the forward region under some specific conditions, known as Kerker’s conditions^24,29–35^. When orthogonal electric and magnetic dipoles of the same amplitude, oscillate in phase^36^ (the first electric and magnetic Mie coefficients *a*_1_, *b*_1_ verify *a*_1_ = *b*_1_) (resp. out of phase^37^ (*a*_1_ = −*b*_1_)), the incident radiation is scattered in the forward (resp. backward) direction, with null (resp. almost null) scattering in the backward (resp. forward) direction for the First Kerker’s condition or Zero-Backward condition (resp. Second Kerker’s condition or near Zero-Forward condition). Recently, more complex structures like metallo-dielectric or dielectric-dielectric isolated core-shell nanoparticles^38–46^ have been investigated for improving the scattering directionality properties established by Kerker *et al*.^29^.

Although the usefulness of HRID isolated particles has been recently proposed for light trapping applications^22^, here we introduce the possibility of using a dimer of HRID particles as an elementary unit for improving the performance of solar cells. Indeed, aggregates of HRID nanoparticles have been analyzed for redirecting the incident radiation into some specific directions eventually different from the forward and backward ones. In particular, they have shown their utility for building optical switching devices^47–51^.

In the following, we demonstrate how, by using HRID dimers under the dipolar approximation, it is possible to find two spectral regions where the incident radiation is preferentially scattered in the forward direction, i.e. in the direction of a potential photosensitive substrate. These spectral regions correspond to the classical Zero-Backward condition (also observed for either isolated or a particle cluster, when there is no electromagnetic interaction between its components) and to a new “near Zero-Backward” condition (originated by the interaction effects between the particles, in our case, a dimer), which is a 180° “rotated” version of the single particle near Zero-Forward condition. In previous works, the possibility of reversing the scattering diagram has been shown by means of more complex configurations^52,53^. In ref.^52^, a metallic trimer was used. When one of the particles of the cluster is shifted with respect to the other two, the interference conditions are changed, leading to a rotation in the scattering diagrams. However, both the metallic character of the particles and the difficulty to get the geometrical arrangement limit its utility. In ref.^53^, the scattering direction can be toggled by a rotation of the polarization of the incident radiation. Nevertheless, the scattered intensity for one of the polarizations is considerably lower than for the other one. Furthermore, in order to observe this effect, multipolar orders have to be included^25^. It is necessary to remark that Kerker’s conditions are only defined for isolated particles. For that reason, in this research we use a more general term: Scattering Directionality Conditions (SDCs), for referring to the Zero-Backward and near Zero-Forward conditions for the dimer configuration.

In the following, we analyze theoretically and experimentally the evolution of the SDCs for a HRID dimer as a function of the distance between the particles (gap), i.e. as a function of their mutual electromagnetic interaction. Notice that, although the experimental measurements have been performed in the microwave region, the results and conclusions of this research can be translated to other spectral regions (VIS-NIR) due to the scalability of the Maxwell’s equations.

The paper is organized as follows: Firstly, we analyze the spectral behavior of the scattered intensity in the forward and backward directions for a dimer of HRID spherical particles for different gaps and polarizations of the incident radiation. Secondly, we show the scattering diagrams at the wavelengths corresponding to both SDCs, for the gaps and polarizations cases studied in the previous section. Finally, we show a brief discussion of the results and a description of the used numerical and experimental methodology.

## Results

### Experimental configuration

**Figure 1:**
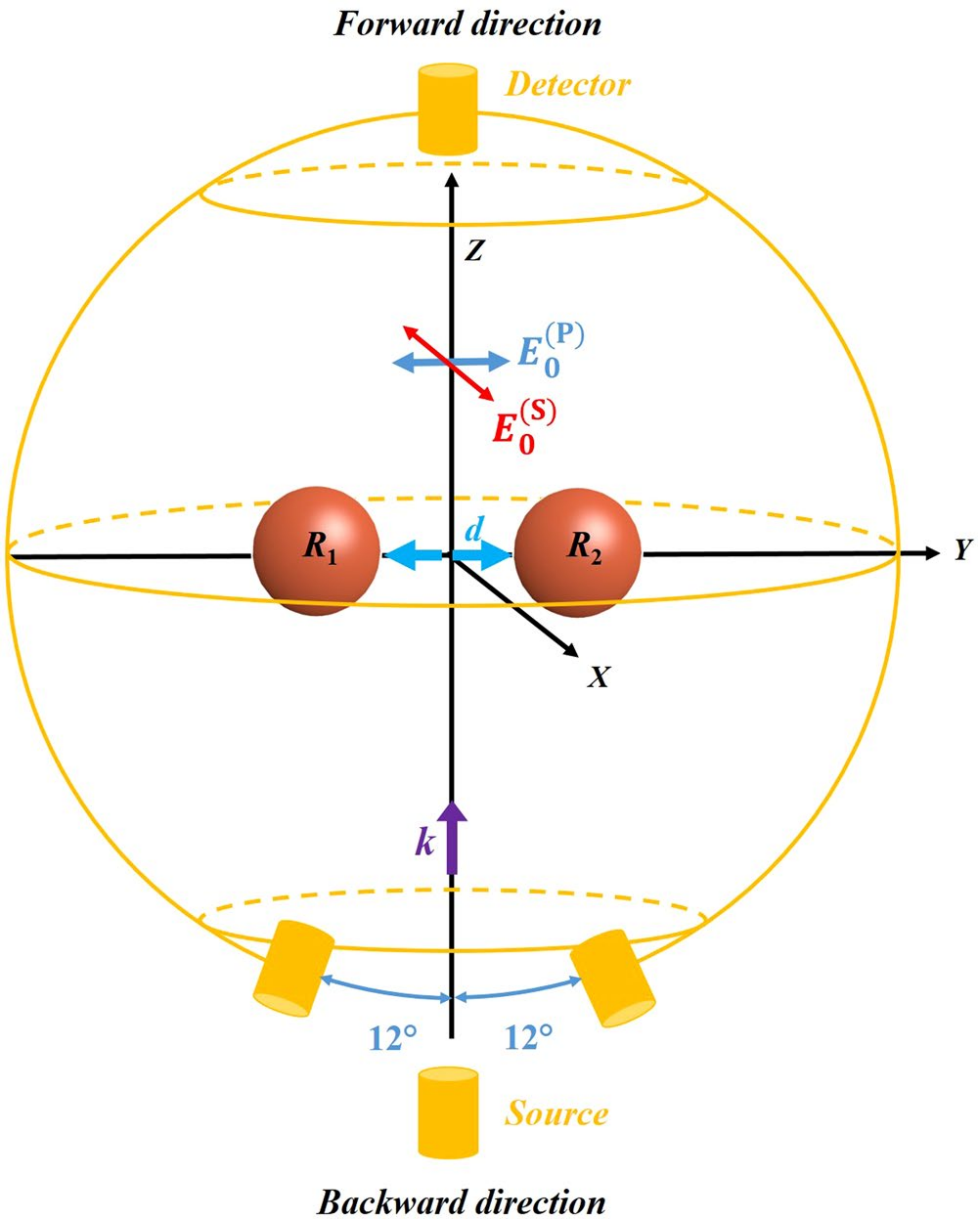
Schematic of the experimental setup. A homogeneous dimer of spherical particles of radius *R*_1_ = *R*_2_ = *R* = 9 mm separated by a gap *d* is illuminated by a plane wave propagating along the *z* axis (***k***) and linearly polarized along the *y* or *x* axis for longitudinal (${{\boldsymbol{E}}}_{0}^{({\rm{P}})}$) or transverse (${{\boldsymbol{E}}}_{0}^{({\rm{S}})}$) configurations, respectively. The yellow circle in the scattering plane (*ZY*) represents the detectors movement. Due to mechanical constraints in the experimental device (see Methods), there is a 24° angle where measurements are unreachable.

SDCs have been studied for a homogeneous dimer of HRID spherical particles smaller than the wavelength of the incident radiation. We define the dimensionless size parameter *q* as 2*πR*/*λ*, where *R* is the particle radius (*R*_1_ = *R*_2_ = *R* = 9 mm) and *λ* is the wavelength of the incident radiation. The electric permittivity has been experimentally determined to be *ε*_1_ = *ε*_2_ = *ε* = 15.7 + 0.3*i* in the analyzed spectral range (*q* = 0.60–1.20), which mimics that of silicon in VIS-NIR. The structure is illuminated by a plane wave propagating along the *z* axis and linearly polarized along either the *y* axis (longitudinal configuration, *P*-incident polarization) or *x* axis (transverse configuration, *S*-incident polarization). The two particles are aligned along the *y* axis. The scattering plane corresponds to *ZY*. A scheme of the experimental setup is in Fig. 1. The gap distance between the two particles is denoted as *d*. We introduce a dimensionless parameter *d*_0_ = *d*/*R*, indicative of the strength of the electromagnetic interaction between the components of the dimer. In this work, we have considered two different gaps: *d*_0_ = 2 and 1/3. For the former, the interaction between the particles is weak and the results resemble those of an isolated particle. For the latter, the interaction between the particles is responsible for the appearance of new specific SDCs, which will be detailed later on. The value *d*_0_ = 1/3 has not been selected at random but comes from a previous observation^49^.

### Intensity scattered by a HRID dimer in the forward and backward directions

The directional properties of the scattered intensity by isolated HRID particles can be understood by means of the coherence effects between the dipolar electric and magnetic resonances. In particular, it has been theoretically^29^ and experimentally^30^ demonstrated how the scattered intensity can be concentrated in the forward or backward directions, evidencing Kerker’s conditions. In this section, we analyze the distribution of the scattered intensity in those specific directions for a HRID dimer.

**Figure 2:**
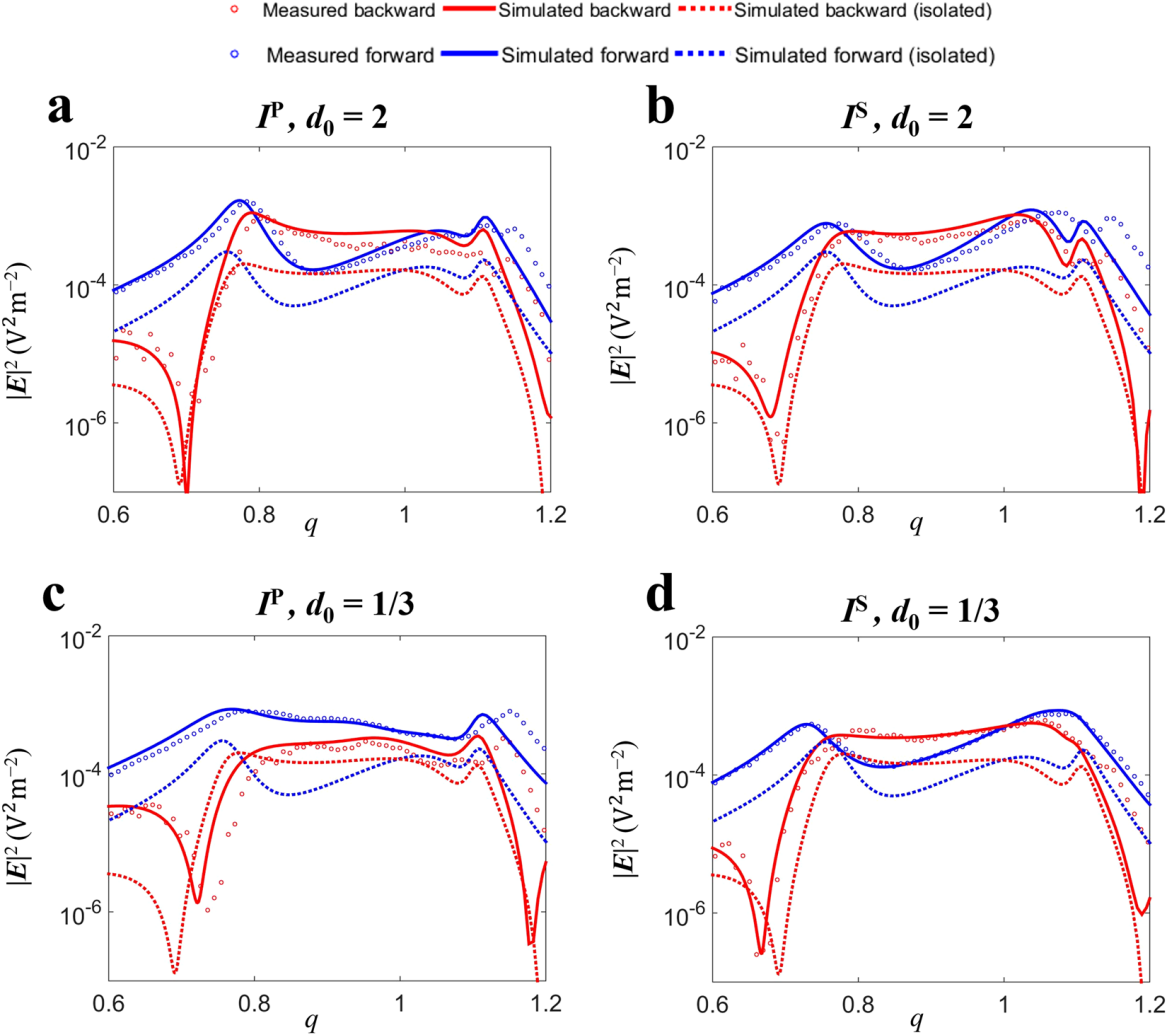
Intensity spectral measurements. Spectral scattered intensity in the forward and backward directions in logarithmic scale for longitudinal (*I*^P^, left column) and transverse (*I*^S^, right column) configurations. (**a**,**b**) Simulation and measurement for *d*_0_ = 2. (**c**,**d**) Simulation and measurement for *d*_0_ = 1/3. For comparison, we show the simulated spectrum corresponding to an isolated particle of radius *R* = 9 mm. Dimer: Simulated backward intensity (solid red line) and measured backward intensity (red circles). Simulated forward intensity (solid blue line) and measured forward intensity (blue circles). Isolated particle: Simulated backward intensity (dotted red line). Simulated forward intensity (dotted blue line).

In Fig. 2 we show, both numerically and experimentally, the spectra corresponding to the scattered intensity in the forward and backward directions for longitudinal (*I*^P^) and transverse (*I*^S^) configurations and for two different gap values: *d*_0_ = 2 (weak interaction) and *d*_0_ = 1/3 (strong interaction). It is worth pointing out that the agreement between the numerical and experimental results is remarkable. In order to establish comparisons, we also plot the case corresponding to an isolated particle of radius *R* = 9 mm.

Due to experimental constraints (see Methods), it is not possible to take measurements in the exact backward direction. Experimentally, the backward (resp. forward) direction corresponds to 12° (resp. 180°). Through simulations, we have compared the scattered intensity at 0° and 12° obtaining almost identical plots. For that reason, in the simulated results, we show the precise backward direction (0°).

Through the spectral response, it is possible to determine the size parameter values where the SDCs hold. Indeed, for *I*^P^ (Fig. 2a,c), around *q* = 0.70 the intensity is mainly scattered in the forward direction and the backward scattered intensity is negligible. This situation corresponds to a SDC (equivalent to a Zero-Backward condition) and is attained independently of the gap. However, as the gap decreases, this condition is blue-shifted (shifting from *q* = 0.70 for *d*_0_ = 2 to *q* = 0.72 for *d*_0_ = 1/3) and the forward scattered intensity increases. Around *q* = 0.87 for *d*_0_ = 2 (Fig. 2a), the incident intensity is mostly backward scattered, but the forward scattered intensity is almost null, which corresponds to another SDC (equivalent to a near Zero-Forward condition). However, as the interaction between the particles increases, this behavior changes drastically. In fact, for *d*_0_ = 1/3 (Fig. 2c), the near Zero-Forward condition is no longer achieved and, at around *q* = 0.87, the intensity is essentially scattered in the forward direction.

The spectra corresponding to the scattered intensity *I*^S^ (*S*-incident polarization) in the forward and backward directions are similar to each other independently of the gap. This is shown by comparing Fig. 2b (*d*_0_ = 2) and 2d (*d*_0_ = 1/3). Contrarily to the longitudinal configuration, as the gap decreases, the Zero-Backward SDC is slightly red-shifted (shifting from *q* = 0.68 for *d*_0_ = 2 to *q* = 0.67 for *d*_0_ = 1/3). The near Zero-Forward condition (which is also red-shifted as the gap is smaller) is attained at around *q* = 0.86 for *d*_0_ = 2 and *q* = 0.84 for *d*_0_ = 1/3. In addition, for *I*^S^, both SDCs are observed at longer wavelengths than for *I*^P^.

When comparing the isolated and dimer spectra, we find a similar behavior when *d*_0_ = 2, because the interaction is weak. Concerning the size parameter values where the SDCs are observed, they are comparable for the dimer and for the isolated particle.

Apart from the tiny spectral shifts, one of the most remarkable results is that it is possible to get a new SDC. This holds only for *P*-incident polarization (*I*^P^) and for strong electromagnetic interaction between the dimer components. We believe that this interesting effect can have great potentiality for photovoltaic applications like solar cells. For either weak electromagnetic interaction cases or *S*-incident polarization (*I*^S^) coupled with strong particle interaction, the electromagnetic behavior resembles that of an isolated particle. As such, at the Zero-Backward condition, the incident radiation is redirected forward, with a null backscattered intensity, which is the desired effect for enhancing solar cells efficiency. However, at the near Zero-Forward condition, the incident intensity is mostly scattered backward, being almost null at forward.

Interestingly, for *P*-incident polarization (*I*^P^) and *d*_0_ = 1/3, only the Zero-Backward condition is attained. In fact, at the size parameter values where the near Zero-Forward condition is observed for the weak interaction case, for *d*_0_ = 1/3, a “near Zero-Backward” condition is found. This means that the incident intensity is mainly scattered in the forward direction, being almost null in backward. Therefore, by using a HRID dimer with strong electromagnetic interaction as an elementary optical building block, it is possible to find two different spectral regions where the incident intensity is mainly redirected in the forward direction. This “enforced” forward scattering with small gaps can have implications in the performance optimization of photovoltaic devices. In order to know the possibility of using these scattering units for improving the efficiency of solar cells, it is necessary to analyze the fraction of incident radiation that is scattered into the substrate (*f*_subs_)^1–3,22^, when the dimer is located on their photosensitive surface. Through the shown results, it is clear that, for *P*-incident polarization, working with a dimer provides higher values of *f*_subs_ than with an isolated particle. However, for *S*-incident polarization, similar results can be obtained independently of the scatterer geometry. Due to solar light is unpolarized, it is necessary to make a balance of *f*_subs_ for different incident polarizations. This analysis leads to a gain in *f*_subs_ when the dimer configuration is considered.

To provide quantitative elements, we compute (in Table 1) the ratio of the scattered directivity between backward and forward directions (*D*_B_/*D*_F_) at the wavelengths where the near Zero-Forward condition is observed. This ratio has been computed for the exact *q* values where that SDC is achieved for each analyzed configuration. We notice that for all the cases, apart for *P*-incident polarization and *d*_0_ = 1/3, the ratio is similar to that obtained for the isolated particle and takes values greater than 1. In fact, the scattered intensity in the backward direction is approximately 3 times bigger than the one scattered in the forward direction. However, for *P*-incident polarization and *d*_0_ = 1/3, the ratio is less than 1, showing indeed that most of the incident radiation is scattered in the forward direction due to the appearance of the new SDC (“near Zero-Backward” condition). This condition, which is a 180° “rotated” version of the traditional near Zero-Forward condition (as we will see by means of the scattering diagrams in the next section), is due to the interaction effects between the particles. In particular, it is caused by the interference of the broad electric and the sharp magnetic dipolar modes, giving rise to a Fano-like resonance. The broadening of the dipolar electric resonance is due to the interaction effects between the electric dipoles in both particles of the dimer (see Supplementary Note 1 for additional details). It should be noticed that the scattered directivity was considered for a collection angle of 30° around 180° and 0° for forward (*D*_F_) and backward (*D*_B_) directions, respectively. This collecting angle was chosen for mimicking the numerical aperture of an optical detection device.

**Table 1:** Directivity values and ratios.

Polarization	Isolated	Dimer (*d*_0_ = 2)		Dimer (*d*_0_ = 1/3)	
	*P/S polar*.	*P polar*.	*S polar*.	*P polar*.	*S polar*.
*D*_B_	2.29	4.99	4.84	1.13	3.37
*D*_F_	0.71	1.41	1.34	2.37	1.12
*D*_B_ */D*_F_	3.20	3.55	3.60	0.48	3.01

The backward *D*_B_ and forward *D*_F_ scattered directivities in far-field as well as their ratio (*D*_B_/*D*_F_) are provided for an isolated particle and for a dimer at the size parameter values where the near Zero-Forward condition is achieved. For the dimer case, the scattered directivity has been computed for both polarizations of the incident radiation (*P*-incident polarization and *S*-incident polarization) and for two interaction parameters *d*_0_ = 2 (weak interaction) and *d*_0_ = 1/3 (strong interaction).

**Figure 3:**
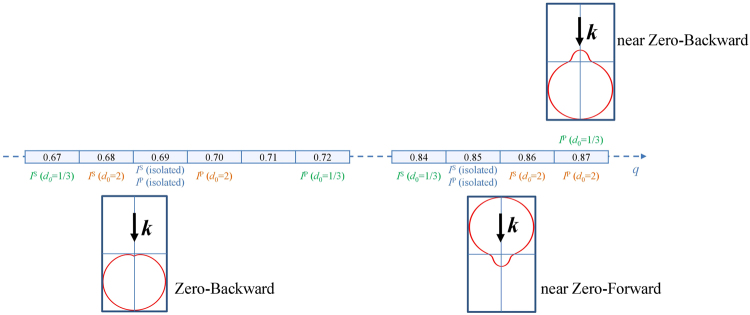
Scheme of the SDCs observed for HRID isolated particles or dimers. The size parameter values where the SDCs are achieved for different polarizations of the incident radiation and distances between the particles (gap) are shown. The black arrow represents the propagation direction of the impinging radiation.

The conclusions of this section are schematically summarized in Fig. 3, where we represent the observed SDCs for a dimer and for an isolated HRID particle.

From Fig. 3, the Zero-Backward condition is observed for all the analyzed cases. However, the near Zero-Forward condition is no longer achieved for *I*^P^ and *d*_0_ = 1/3. For this configuration, a “near Zero-Backward” condition manifests.

### Scattered intensity diagrams

**Figure 4:**
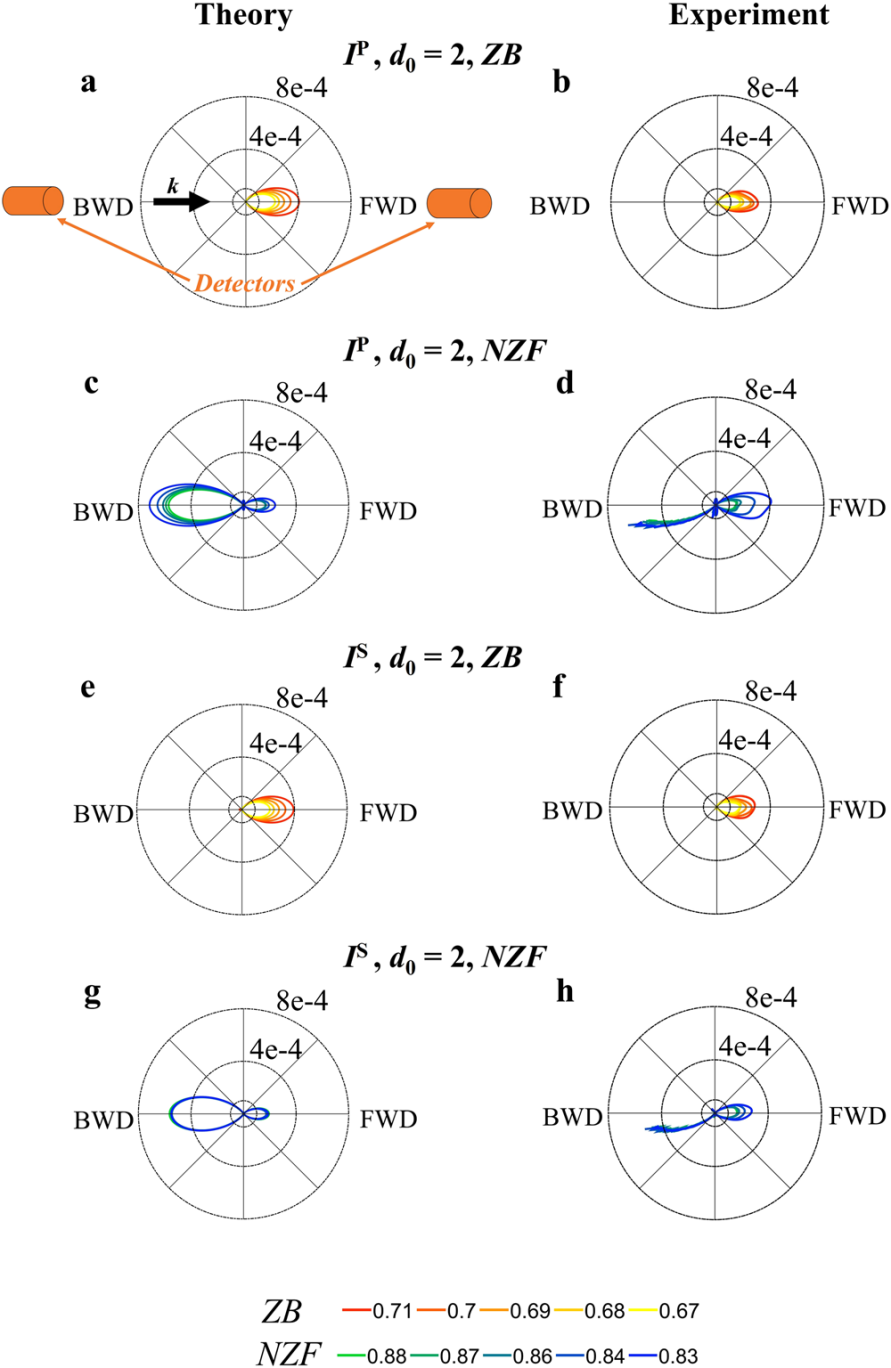
Angular variations of scattered intensities. Scattering intensity diagrams in the scattering plane (*ZY*, see Fig. 1) at the frequencies where the SDCs are observed for a HRID dimer (*d*_0_ = 2). In the first (**a**,**b**) and third (**e**,**f**) rows, we show the diagrams corresponding to the Zero-Backward (*ZB*) condition (*q* = 0.71, 0.70, 0.69, 0.68, 0.67) for longitudinal and transverse configurations, respectively. In the second (**c**,**d**) and fourth (**g**,**h**) rows, we plot the scattering diagrams at the near Zero-Forward (*NZF*) condition (*q* = 0.88, 0.87, 0.86, 0.84, 0.83) for *P*-incident polarization and *S*-incident polarization, respectively. See bottom lines for color codes. Left and right columns correspond to simulations and experimental measurements, respectively. The black arrow represents the direction of the incident radiation (***k***). The orange cylinders indicate the detectors positions. FWD stands for Forward direction and BWD stands for Backward direction. The measured intensities are presented for the angles between 12° and 180°, the angles beyond 12° being unreachable due to mechanical constraints in the measurement device.

**Figure 5:**
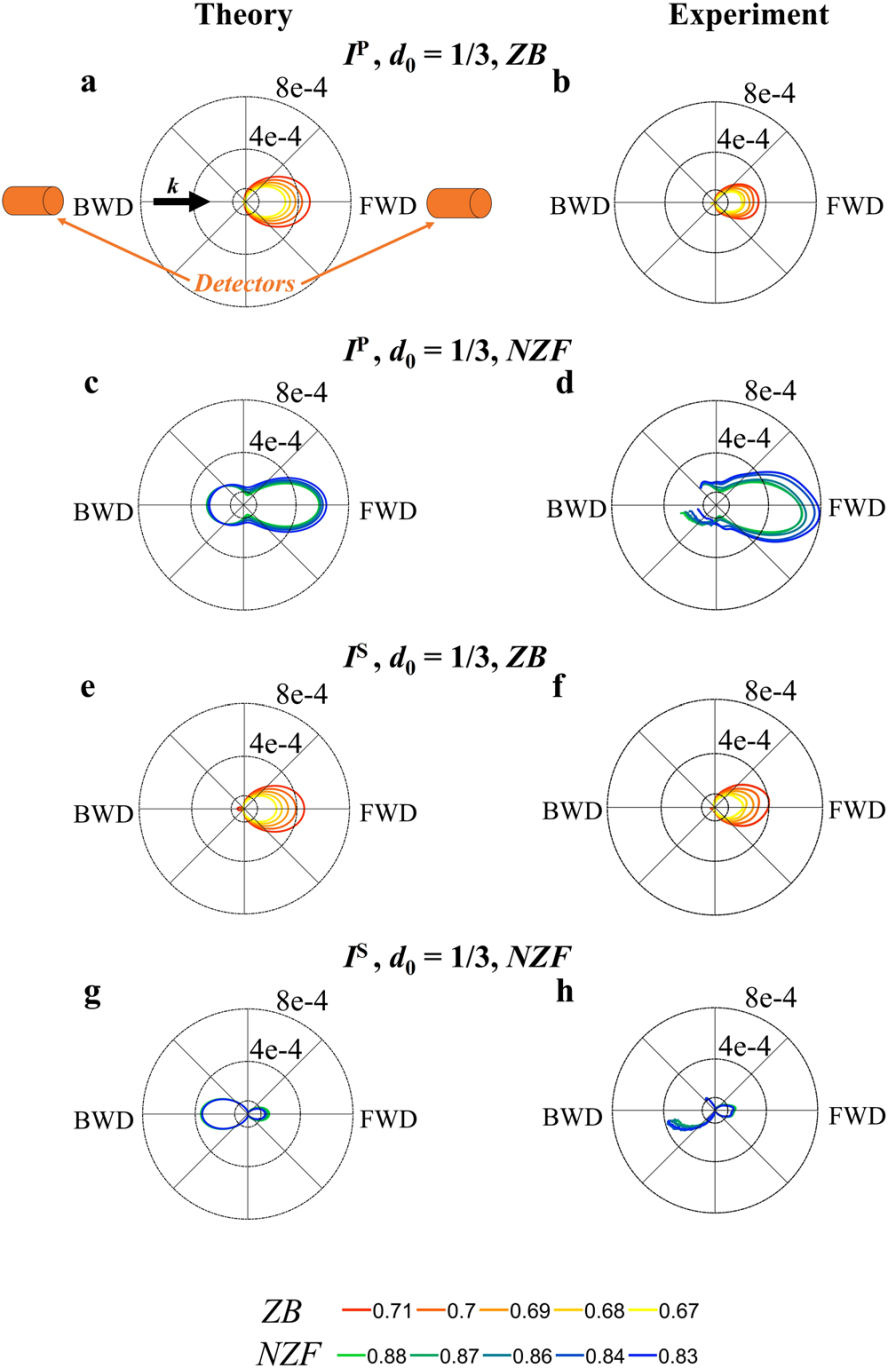
Angular variations of scattered intensities. Scattering intensity diagrams in the scattering plane (*ZY*, see Fig. 1) at the frequencies where the SDCs are observed for a HRID dimer (*d*_0_ = 1/3). In the first (**a**,**b**) and third (**e**,**f**) rows we show the diagrams corresponding to the Zero-Backward (*ZB*) condition (*q* = 0.71, 0.70, 0.69, 0.68, 0.67) for longitudinal and transverse configurations, respectively. In the second (**c**,**d**) and fourth (**g**,**h**) rows we plot the scattering diagrams at the near Zero-Forward (*NZF*) condition (*q* = 0.88, 0.87, 0.86, 0.84, 0.83) for *P*-incident polarization and *S*-incident polarization, respectively. See bottom lines for color codes. Left and right columns correspond to simulations and experimental measurements, respectively. The black arrow represents the direction of the incident radiation (***k***). The orange cylinders indicate the detectors positions. FWD stands for Forward direction and BWD stands for Backward direction. The measured intensities are presented for the angles between 12° and 180°, the angles beyond 12° being unreachable due to mechanical constraints in the measurement device.

To confirm the “rotation” effect of the scattering pattern, we analyze the angular distribution of the scattered intensity in far-field for a dimer of HRID particles at the frequencies where the SDCs appear. In Figs 4 and 5 we show (numerically and experimentally) the scattering diagrams in the scattering plane (*ZY*, see Fig. 1) for the interaction parameters *d*_0_ = 2 and 1/3, respectively. Due to the experimental constraints (see Methods), the experimental diagrams are shown for angles between 12° and 180°. The first thing to notice is that the experimental scattering diagrams are in full agreement with those obtained numerically.

**Figure 6:**
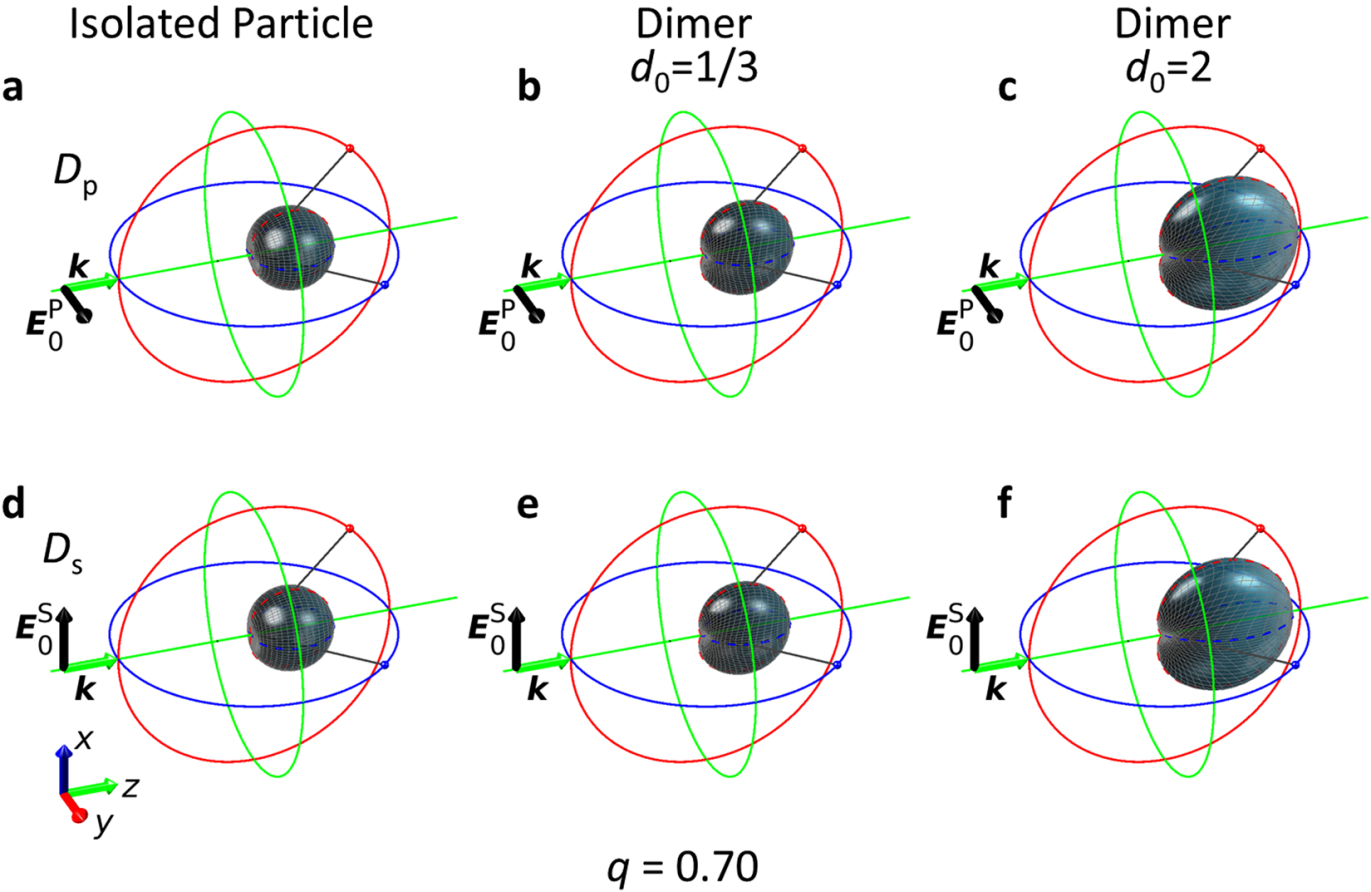
3D calculated scattering directivity diagrams for an isolated sphere and for a dimer at *q* = 0.70. The structure is illuminated with a plane wave propagating along the *z* axis (***k***) and linearly polarized parallel to the *y* axis (${{\boldsymbol{E}}}_{0}^{{\rm{P}}}$) (longitudinal configuration) or to the *x* axis (${{\boldsymbol{E}}}_{0}^{{\rm{S}}}$) (transverse configuration), first and second rows, respectively. Isolated particle: (**a**,**d**). Strong interaction between the dimer components (*d*_0_ = 1/3) (**b**,**e**). Weak interaction between both particles of the dimer (*d*_0_ = 2) (**c**,**f**).

**Figure 7:**
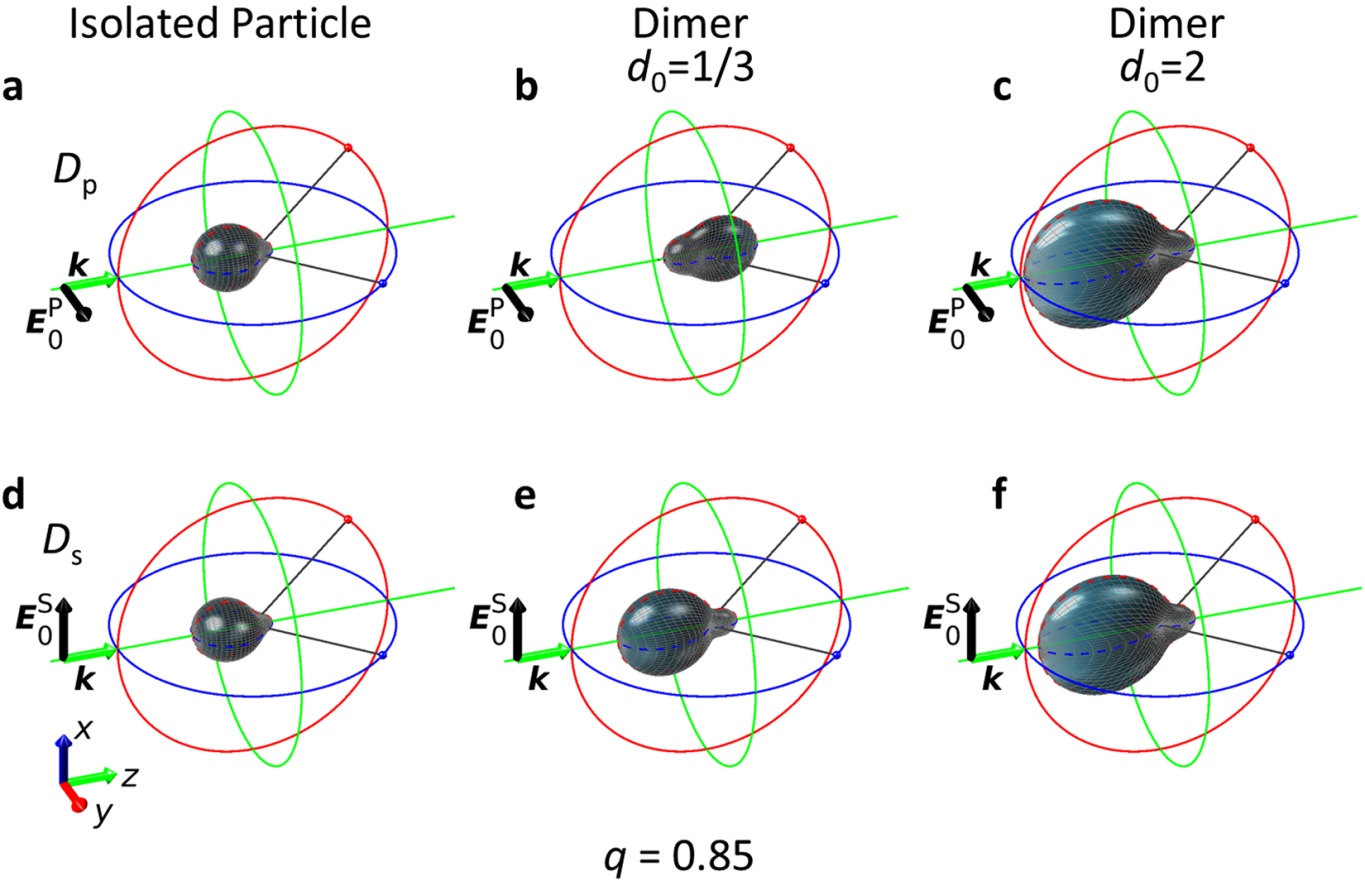
3D calculated scattering directivity diagrams for an isolated sphere and for a dimer at *q* = 0.85. The structure is illuminated with a plane wave propagating along the *z* axis (***k***) and linearly polarized parallel to the *y* axis (${{\boldsymbol{E}}}_{0}^{{\rm{P}}}$) (longitudinal configuration) or to the *x* axis (${{\boldsymbol{E}}}_{0}^{{\rm{S}}}$) (transverse configuration), first and second rows, respectively. Isolated particle: (**a**,**d**). Strong interaction between the dimer components (*d*_0_ = 1/3) (**b**,**e**). Weak interaction between both particles of the dimer (*d*_0_ = 2) (**c**,**f**).

For the sake of completeness, in Figs 6 and 7, we also present the 3D scattering directivity diagrams calculated analytically by means of the Green’s formalism for the different dimer configurations and for an isolated particle. The scattering diagrams in the scattering plane for the isolated particle can be found in Supplementary Note 2.

When the interaction effects between the particles are weak, at *q* around 0.70 (resp. *q* around 0.85) the incident radiation is mainly scattered in the forward (resp. backward) direction, and is null (resp. almost null) in the backward (resp. forward) one. It corresponds to a Zero-Backward (resp. near Zero-Forward) condition. These observed SDCs, which are the same as those for an isolated particle, are attained whatever the polarization of the incident radiation is (either *S* or *P*). However, for strong interaction effects, the electromagnetic response is different depending on the polarization of the impinging radiation. For the transverse configuration (*S*-incident polarization), the SDCs are similar to those corresponding to weak interaction effects, i.e. Zero-Backward and near Zero-Forward conditions are observed. Nevertheless, for the longitudinal configuration (*P*-incident polarization), only the Zero-Backward condition is preserved. For size parameter values where the near Zero-Forward condition was found for the other analyzed cases, for this specific configuration, the intensity is mainly scattered in the forward direction, being almost null in backward, this is the reason for the “near Zero-Backward” condition denomination. By comparing the scattering diagrams at *q* around 0.85, it is obvious that the “near Zero-Backward” condition (see Fig. 5c,d) is a “rotation” of the conventional near Zero-Forward condition (see Figs 4c,d,g,h and 5g,h).

It is important to remark that the scattering patterns show a smooth variation when the frequency of the incident radiation is slightly modified. In all the analyzed cases, only one lobe is observed. The fact that the SDCs do not change drastically with the size parameter is significant from an experimental point of view. Small uncertainties in the particle radius or in the impinging frequency will provide almost identical results.

When comparing the scattering diagrams for *d*_0_ = 2 (see Fig. 4) and *d*_0_ = 1/3 (see Fig. 5), it is evident that they are more directional (narrower in angular spread) for the weak interaction case. For both analyzed values of the gap, the angular distribution of the scattered intensity is narrower for the dimer than for the isolated particle^28,54–57^. Also, the amount of scattered intensity is larger for the dimer structure. This is due to two different effects. On one hand, the incident field is collected by two particles instead of one. On the other, the interaction between the particles give rise to constructive interferences zones. As the dimer is bigger than the isolated particle compared to the wavelength, this leads to a narrower lobe in the radiation pattern.

## Discussion

A scattering unit consisting of a homogeneous dimer of HRID spherical particles for redirecting the incident radiation in the forward direction has been introduced theoretically and experimentally. It has also been shown that this is more efficient than the isolated particle unit. Recently, isolated HRID particles have been proposed as an alternative to metallic ones for optimizing the performance of thin film solar cells^22^. By means of the analysis of the SDCs for a HRID dimer as a function of the distance between the particles (gap), i.e. as a function of their electromagnetic interaction, we have demonstrated that in the spectral range where the dipolar approximation holds, two spectral regions where the intensity is mainly scattered in the forward direction can be found. They correspond to the Zero-Backward condition (also put into evidence for isolated particles) and to a “near Zero-Backward” condition, which is a “rotated” version of the near Zero-Forward condition, whose origin is the interaction effects between both components of the dimer. Therefore, a HRID dimer, with control of its gap distance, presents two main advantages with respect to the isolated particle case:

1. The near Zero-Forward condition is no longer observed. In fact, for strong interaction effects between the particles (at the frequencies where that condition holds in the case of weak interacting dimers) a “near Zero-Backward” condition can be observed, where the incident radiation is mainly scattered forward.
2. The Zero-Backward condition can be observed independently of the distance between the particles of the dimer (gap). Under these conditions, we can find a broader spectral region where the incident radiation is preferentially scattered in the forward direction. This can have some interesting implications for boosting the efficiency of photovoltaic devices, which require broadband response for redirection of the incident radiation into the photosensitive surface.

Another application of this kind of HRID structures is in metasurfaces. In particular, a dimer of HRID particles was proposed as an elementary unit for building metasurfaces, which can work as a high reflector or a high transmitter depending on the polarization of the incident radiation^58^.

The dimer directionality properties may be altered by the presence of the substrate (photosensitive). In our future work, we plan to analyze the scattered intensity into the photovoltaic layer when a HRID dimer of spherical particles is located on its surface.

In recent works^59,60^ it was demonstrated that absorption directivity is required in order to provide voltage enhancements and, consequently, large efficiencies of solar cells. For that reason, voltage enhancements produced by the directionality properties of our proposed configuration should be assessed in future research.

Finally, it is important to remark that, in spite of the fact that the experimental validation of our proposed dimer model has been carried out in the microwave domain (frequency in the GHz range), due to the scalability of the Maxwell’s equations, the results shown in this research are fully re-scalable to other frequency ranges and dimension scales, for instance, the near-infrared (λ ~ 1000–2000 nm) and the nanometric scale (*R* ~ 200 nm). Also, the spheres used in our experiment are made of a material (HIK 500 F from Laird Technologies, see Methods), whose optical properties mimic those of Silicon in the NIR.

## Methods

### Numerical methods

Parts of the numerical results have been obtained by means of COMSOL Multiphysics^61^. This commercial software is based on the Finite Element Method. In particular, for carrying out the results we used the Radiofrequency Module, which allows us to formulate and solve the differential form of Maxwell’s equations (in the frequency domain) together with boundary conditions. Both particles were embedded in the center of a vacuum sphere of radius *λ*/2 + 2 *R*. The air sphere is surrounded by a perfectly matched layer (PML) of thickness *λ/*4, which acts as an absorber of the scattered field. The mesh was chosen fine enough for allowing the convergence of the results. In particular, the element size of the mesh of the embedding medium is smaller than *λ/5* and that of the particles is smaller than *λ/*3*Re(n)*, *n* being the refractive index of the particle.

The 3D scattering directivity diagrams were obtained with a Green function formalism in the spectral range where the dipolar approximation holds. An electric dipole and a magnetic dipole are positioned at the center of each HRID sphere and their amplitude is adapted in order to take into account the coupling effects. For each polarization case, the radiation intensity *I* is computed and normalized by the total radiated power *I*_tot_ to provide the 3D scattering directivity diagrams, i.e., *D* = 4 π *I*/*I*_tot_.

### Experimental methods

**Figure 8:**
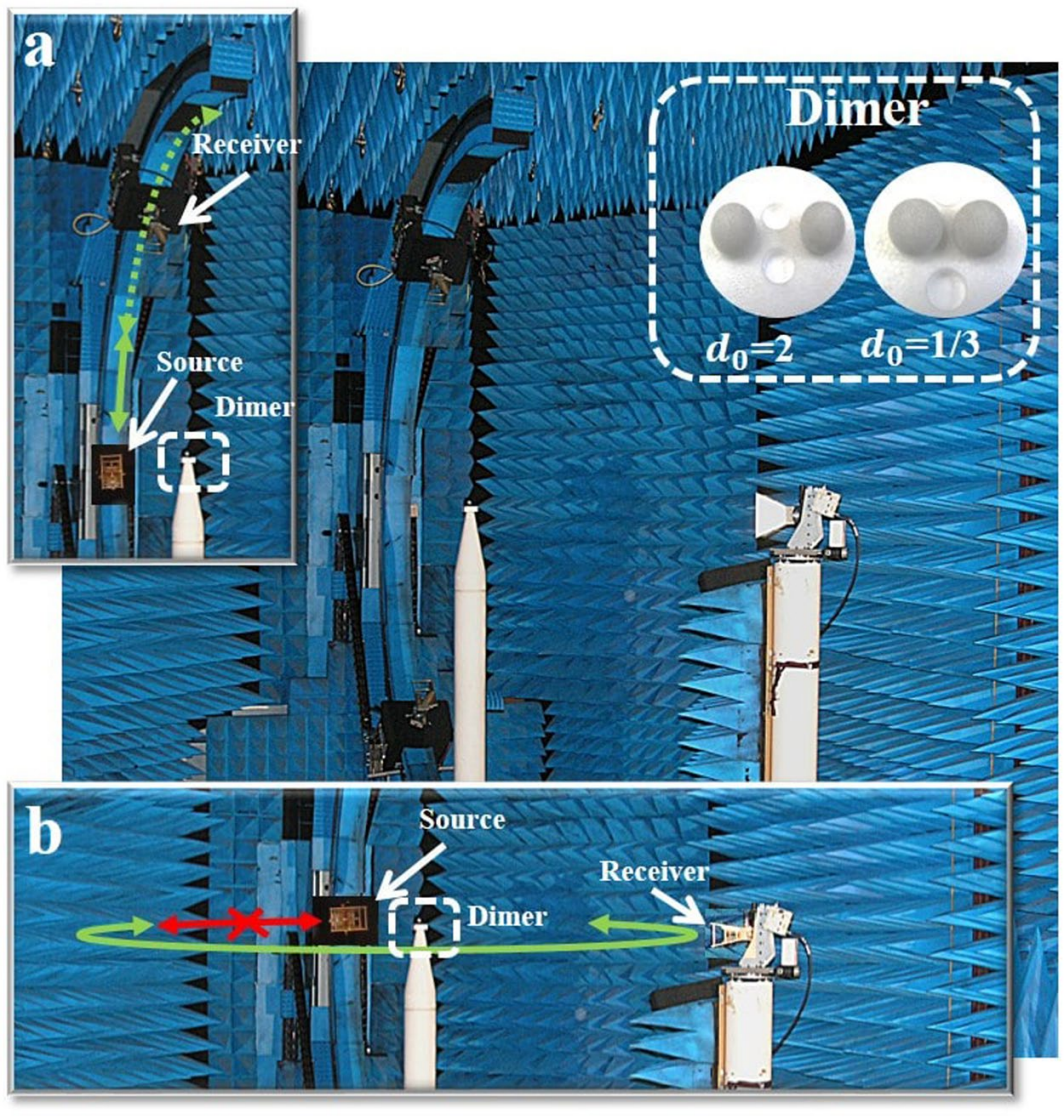
Photography of the experimental setup in the anechoic chamber of the CCRM. The dimer is placed at the top of a polystyrene mast and is excited by a source antenna emitting a linearly polarized wave (parallel or perpendicular to the dimer principle axis). The configuration in (**a**) is used to acquire scattering angles between 12° and 50°, where the two HRID spheres are placed on the mast, one on top of the other with the desired gap between them, and the receiver moves circularly around them in a vertical trajectory. The configuration in (**b**) is used to acquire scattering angles between 50° and 310°, where the two HRID spheres are placed on the mast, one next to the other with the desired gap between them, and the receiver moves circularly around them in a horizontal trajectory. As an inset, the two HRID spheres in both large- and small-gap cases are shown (*d*_0_ = 2 and *d*_0_ = 1/3, respectively).

The experimental measurements were carried out at microwave frequencies in an anechoic chamber at the Centre Commun de Ressources en Microondes (CCRM) in Marseille, France. We take advantage from the scalability of the Maxwell’s equations to re-scale the measurements from the optical domain to the microwave domain, by increasing both the wavelength of the illumination and the diameter of the particles, but while keeping fixed the ratio between them and the same optical properties. Therefore, the measurement results could be similarly obtained in the VIS-NIR range by using silicon (Si) spherical particles of radius of hundreds of nanometers. This facility has interestingly become a specialized microwave scattering device to perform analog to light measurements on a variety of complex particles^62^. The scattering measurements on a single HRID subwavelength sphere have recently been implemented to experimentally demonstrate the directional tunability of scattering radiations^25^ and allowed the experimental proof of the Kerker’s conditions^30^. In this work, we have used this experimental setup for measuring the scattered intensity by a homogenous dimer of HRID spherical particles. The HRID dimer is placed on top of a polystyrene mast at the center of a circular setup of 4 meters diameter. It is illuminated with a linearly polarized plane wave generated by a fixed position source antenna (Fig. 8). Two geometrical configurations are used to measure the scattered intensity. The first configuration allows the measurement for scattering angles between 12° and 50° and it uses the displacement of the receiver on a vertical arch (Fig. 8a). The second configuration allows to measure the scattered intensity for scattering angles between 50° and 310°. It uses the displacement of the receiving antenna in the horizontal plane containing the source, the receiver and the dimer (Fig. 8b). Measurements over scattering angles below 12° are unreachable to avoid a too strong coupling between the antennas and a mechanical collision between the source and the receiver carriages. More details about the two configurations can be found in ref.^30^. Both polarizations, corresponding to transverse and longitudinal configurations, are used and the switch between them is simply achieved by rotating the source and receiving antennas. The homogeneous dimer is formed by two particles of radius *R*_1_ = *R*_2_ = 9 mm. They are made of a HRID material (HIK 500 F from Laird Technologies). Their electric permittivity is *ε* = 15.7 + 0.3i and has been experimentally estimated from far field scattering measurements.

To characterize the optical properties of the particles, the field scattered by a single spherical homogeneous particle is measured. The complex permittivity of the particle is chosen as the one corresponding to the best fit, in the least square sense, between Mie computations and the measured field, taking into account the measurement standard deviations. More details can be found in ref.^63^.

The distance between the particles (gap) was controlled by means of a polystyrene holder where spheres can be located at different distances from each other, as it is observed through the inset of Fig. 8.

## Electronic supplementary material

Supplementary Information
