# Supplementary material for: On the scattering directionality of a dielectric particle dimer of High Refractive Index
Source: Sci Rep. 2018 May 22;8:7976. doi: 10.1038/s41598-018-26359-8 (PMC5964075; doi:10.1038/s41598-018-26359-8)
Supplement: Supplementary file 1 — Supplementary Information [file 41598_2018_26359_MOESM1_ESM.pdf]

# On the scattering directionality of a dielectric particle dimer of High Refractive Index

Ángela I. Barreda[1], Hassan Saleh[2,3], Amelie Litman[2], Francisco González[1], Jean-Michel Geffrin[2] & Fernando Moreno[1,*]

[1]Group of Optics, Department of Applied Physics, University of Cantabria, Cantabria 39005, Spain.

[2]Aix-Marseille Univ, CNRS, Centrale Marseille, Institut Fresnel, Marseille, France.

[3]Centre Commun de Ressources en Microondes CCRM, 5 rue Enrico Fermi, Marseille 13453, France.

[*]corresponding author: morenof@unican.es

**Supplementary Note 1**

**Green's method to model the electric-magnetic dipole interaction.** In this part, in order to understand the underlying physics associated to the "near Zero-Backward" condition, we analytically study the different contributions of both particles to the scattered intensity by means of a Green's function formalism and a dipolar approximation. Following this analytical approach, the expressions of the induced electric and magnetic dipoles in each particle (which are the result of the incident electric and magnetic fields and the fields created by the interaction between both components of the dimer) are given by two sets of coupled equations.

For the $P$-incident polarization, the interaction is detailed in the Supplementary Equations (1-2)

$$\begin{pmatrix} 1 & -\alpha_{\mathrm{e}} k^2 g_{\mathrm{yy}} \\ -\alpha_{\mathrm{e}} k^2 g_{\mathrm{yy}} & 1 \end{pmatrix} \begin{pmatrix} p_{1\mathrm{y}}^{\mathrm{P}} \\ p_{2\mathrm{y}}^{\mathrm{P}} \end{pmatrix} = \begin{pmatrix} \varepsilon_0 \varepsilon_{\mathrm{h}} \alpha_{\mathrm{e}} E_0 \\ \varepsilon_0 \varepsilon_{\mathrm{h}} \alpha_{\mathrm{e}} E_0 \end{pmatrix} \quad (1)$$

and

$$\begin{pmatrix} \begin{bmatrix} 1 & \alpha_{\mathrm{e}} k^2 g_{\mathrm{xx}} \\ \alpha_{\mathrm{e}} k^2 g_{\mathrm{xx}} & 1 \end{bmatrix} & \begin{bmatrix} 0 & -i\varepsilon_0 \varepsilon_{\mathrm{h}} \alpha_{\mathrm{e}} Z k^2 g_{\mathrm{zx}} \\ i\varepsilon_0 \varepsilon_{\mathrm{h}} \alpha_{\mathrm{e}} Z k^2 g_{\mathrm{zx}} & 0 \end{bmatrix} \\ \begin{bmatrix} 0 & -\frac{i\alpha_{\mathrm{m}}}{Z} \frac{k^2}{\varepsilon_0 \varepsilon_{\mathrm{h}}} g_{\mathrm{zx}} \\ \frac{i\alpha_{\mathrm{m}}}{Z} \frac{k^2}{\varepsilon_0 \varepsilon_{\mathrm{h}}} g_{\mathrm{zx}} & 0 \end{bmatrix} & \begin{bmatrix} 1 & \alpha_{\mathrm{m}} k^2 g_{\mathrm{xx}} \\ \alpha_{\mathrm{m}} k^2 g_{\mathrm{xx}} & 1 \end{bmatrix} \end{pmatrix} \begin{pmatrix} p_{1\mathrm{z}}^{\mathrm{P}} \\ p_{2\mathrm{z}}^{\mathrm{P}} \\ m_{1\mathrm{x}}^{\mathrm{P}} \\ m_{2\mathrm{x}}^{\mathrm{P}} \end{pmatrix} = \begin{pmatrix} 0 \\ 0 \\ -\alpha_{\mathrm{m}} \frac{E_0}{Z} \\ -\alpha_{\mathrm{m}} \frac{E_0}{Z} \end{pmatrix} (2)$$

For the *S*-incident polarization, the interaction is detailed in the Supplementary Equations (3-4)

$$\begin{pmatrix} 1 & -\alpha_{\mathrm{m}}k^2 g_{\mathrm{yy}} \\ -\alpha_{\mathrm{m}}k^2 g_{\mathrm{yy}} & 1 \end{pmatrix} \begin{pmatrix} -m_{1\mathrm{y}}^{\mathrm{S}} \\ -m_{2\mathrm{y}}^{\mathrm{S}} \end{pmatrix} = \begin{pmatrix} -\alpha_{\mathrm{m}}\frac{E_0}{Z} \\ -\alpha_{\mathrm{m}}\frac{E_0}{Z} \end{pmatrix} \tag{3}$$

and

$$\begin{pmatrix} \begin{bmatrix} 1 & \alpha_{\mathrm{e}}k^2 g_{\mathrm{xx}} \\ \alpha_{\mathrm{e}}k^2 g_{\mathrm{xx}} & 1 \end{bmatrix} & \begin{bmatrix} 0 & -i\varepsilon_0\varepsilon_{\mathrm{h}}\alpha_{\mathrm{e}}Zk^2 g_{\mathrm{zx}} \\ i\varepsilon_0\varepsilon_{\mathrm{h}}\alpha_{\mathrm{e}}Zk^2 g_{\mathrm{zx}} & 0 \end{bmatrix} \\ \begin{bmatrix} 0 & -\frac{i\alpha_{\mathrm{m}}}{Z}\frac{k^2}{\varepsilon_0\varepsilon_{\mathrm{h}}}g_{\mathrm{zx}} \\ \frac{i\alpha_{\mathrm{m}}}{Z}\frac{k^2}{\varepsilon_0\varepsilon_{\mathrm{h}}}g_{\mathrm{zx}} & 0 \end{bmatrix} & \begin{bmatrix} 1 & \alpha_{\mathrm{m}}k^2 g_{\mathrm{xx}} \\ \alpha_{\mathrm{m}}k^2 g_{\mathrm{xx}} & 1 \end{bmatrix} \end{pmatrix} \begin{pmatrix} p_{1\mathrm{x}}^{\mathrm{S}} \\ p_{2\mathrm{x}}^{\mathrm{S}} \\ -m_{1\mathrm{z}}^{\mathrm{S}} \\ -m_{2\mathrm{z}}^{\mathrm{S}} \end{pmatrix} = \begin{pmatrix} \varepsilon_0\varepsilon_{\mathrm{h}}\alpha_{\mathrm{e}}E_0 \\ \varepsilon_0\varepsilon_{\mathrm{h}}\alpha_{\mathrm{e}}E_0 \\ 0 \\ 0 \end{pmatrix} \tag{4}$$

where $p_{\mathrm{jx}}^{\mathrm{P}}$, $p_{\mathrm{jy}}^{\mathrm{P}}$, $p_{\mathrm{jz}}^{\mathrm{P}}$, $p_{\mathrm{jx}}^{\mathrm{S}}$, $p_{\mathrm{jy}}^{\mathrm{S}}$, $p_{\mathrm{jz}}^{\mathrm{S}}$ are the electric dipole moments induced in the *j*th particle (*j* = 1, 2) along the *x*, *y*, *z* axis for longitudinal and transverse configurations, respectively. $m_{\mathrm{jx}}^{\mathrm{P}}$, $m_{\mathrm{jy}}^{\mathrm{P}}$, $m_{\mathrm{jz}}^{\mathrm{P}}$, $m_{\mathrm{jx}}^{\mathrm{S}}$, $m_{\mathrm{jy}}^{\mathrm{S}}$, $m_{\mathrm{jz}}^{\mathrm{S}}$ are the magnetic dipole moments induced in the *j*th particle (*j* = 1, 2) along the *x*, *y*, *z* axis for longitudinal and transverse configurations, respectively. $\varepsilon_0$ is the dielectric permittivity of the vacuum, $\varepsilon_{\mathrm{h}}$ is the relative dielectric permittivity of the embedding medium, $Z$ is the surrounding medium impedance, $k$ is the wavenumber of the impinging radiation in the embedding medium, $\alpha_{\mathrm{e}}$ and $\alpha_{\mathrm{m}}$ are the electric and magnetic polarizabilities, respectively. $E_0$ is the amplitude of the electric field. $g_{\mathrm{xx}}$, $g_{\mathrm{zx}}$, $g_{\mathrm{yy}}$ are given by Supplementary Equations (5-7).

$$g_{\mathrm{xx}} = -\left\{\left(1 + \frac{i}{kD} - \frac{1}{k^2D^2}\right)\right\} g(D) \tag{5}$$

$$g_{\mathrm{zx}} = -\left(i - \frac{1}{kD}\right) g(D) \tag{6}$$

$$g_{\mathrm{yy}} = \left\{\left(-\frac{2i}{kD} + \frac{2}{k^2D^2}\right)\right\} g(D) \tag{7}$$

*D* being the distance between the center of both spheres, *i.e.*, the distance between the two sets of dipoles. $g(D)=\mathrm{e}^{ikD}/4\pi D$ is the scalar Green's function.

By solving the systems of linear equations (Supplementary Equations (1-2) and (3-4)), it is possible to obtain the components of the resulting induced electric and magnetic

dipoles for longitudinal and transverse configurations, respectively. In order to get the far-field scattered electric field, the induced electric and magnetic dipoles should be introduced into the Supplementary Equations (8) and (9), respectively.

$$\boldsymbol{E}_{\mathrm{p}}^{\mathrm{ff}}(\boldsymbol{r}) \approx \frac{k^2 e^{ik|r-r_{\mathrm{d}}|}}{4\pi\varepsilon_{\mathrm{h}}\varepsilon_0|r-r_{\mathrm{d}}|}[\boldsymbol{p}(\boldsymbol{r}_{\mathrm{d}}) - (\boldsymbol{u}_{\mathrm{r}} \cdot \boldsymbol{p}(\boldsymbol{r}_{\mathrm{d}}))\boldsymbol{u}_{\mathrm{r}}] \tag{8}$$

$$\boldsymbol{E}_{\mathrm{m}}^{\mathrm{ff}}(\boldsymbol{r}) \approx -Z\frac{k^2 e^{ik|r-r_{\mathrm{d}}|}}{4\pi|r-r_{\mathrm{d}}|}[\boldsymbol{u}_{\mathrm{r}} \times \boldsymbol{m}(\boldsymbol{r}_{\mathrm{d}})] \tag{9}$$

where $\boldsymbol{r}$ indicates the detector location, $\boldsymbol{r}_{\mathrm{d}}$ the dipole location and $\boldsymbol{u}_{\mathrm{r}} = \frac{r-r_{\mathrm{d}}}{|r-r_{\mathrm{d}}|}$

$\boldsymbol{E}_{\mathrm{p1}}^{\mathrm{ff,P}}$, $\boldsymbol{E}_{\mathrm{p2}}^{\mathrm{ff,P}}$, $\boldsymbol{E}_{\mathrm{m1}}^{\mathrm{ff,P}}$, $\mathbf{E}_{\mathrm{m2}}^{\mathrm{ff,P}}$, $\boldsymbol{E}_{\mathrm{p1}}^{\mathrm{ff,S}}$, $\boldsymbol{E}_{\mathrm{p2}}^{\mathrm{ff,S}}$, $\boldsymbol{E}_{\mathrm{m1}}^{\mathrm{ff,S}}$ and $\mathbf{E}_{\mathrm{m2}}^{\mathrm{ff,S}}$ are the scattered electric fields in the far-field approximation ($\boldsymbol{E}^{\mathrm{ff}}$) created by the induced electric ($\boldsymbol{E}_{\mathrm{p}}^{\mathrm{ff}}$) and magnetic ($\boldsymbol{E}_{\mathrm{m}}^{\mathrm{ff}}$) dipoles in particle 1 ($\boldsymbol{E}_{\mathrm{p1/m1}}^{\mathrm{ff}}$) and particle 2 ($\boldsymbol{E}_{\mathrm{p2/m2}}^{\mathrm{ff}}$) and for *P*-incident polarization ($\boldsymbol{E}^{\mathrm{ff,P}}$) and *S*-incident polarization ($\boldsymbol{E}^{\mathrm{ff,S}}$). For forward (resp. backward), 180° (resp. 0°), $\boldsymbol{u}_{\mathrm{r}}$ will be assumed to be parallel to the *z* axis, as $|\boldsymbol{r}| >> |\boldsymbol{r}_{\mathrm{d}}|$. The previous equations simplify into Supplementary Equations (10-11) (resp. Supplementary Equations (12-13)) for longitudinal (resp. transverse) configuration, for the particle *j* = 1, 2.

$$\boldsymbol{E}_{\mathrm{pj}}^{\mathrm{ff,P}}(180°) = \boldsymbol{E}_{\mathrm{pj}}^{\mathrm{ff,P}}(0°) \approx \frac{v}{\varepsilon_{\mathrm{h}}\varepsilon_0} p_{\mathrm{jy}}^{\mathrm{P}} \boldsymbol{e}_{\mathrm{y}} \tag{10}$$

$$\boldsymbol{E}_{\mathrm{mj}}^{\mathrm{ff,P}}(180°) = -\boldsymbol{E}_{\mathrm{mj}}^{\mathrm{ff,P}}(0°) \approx -\mathrm{Z}v\, m_{\mathrm{jx}}^{\mathrm{P}} \boldsymbol{e}_{\mathrm{y}} \tag{11}$$

$$\boldsymbol{E}_{\mathrm{pj}}^{\mathrm{ff,S}}(180°) = \boldsymbol{E}_{\mathrm{pj}}^{\mathrm{ff,S}}(0°) \approx \frac{v}{\varepsilon_{\mathrm{h}}\varepsilon_0} p_{\mathrm{jx}}^{\mathrm{S}} \boldsymbol{e}_{\mathrm{x}} \tag{12}$$

$$\boldsymbol{E}_{\mathrm{mj}}^{\mathrm{ff,S}}(180°) = -\boldsymbol{E}_{\mathrm{mj}}^{\mathrm{ff,S}}(0°) \approx \mathrm{Z}v\, m_{\mathrm{jy}}^{\mathrm{S}} \boldsymbol{e}_{\mathrm{x}} \tag{13}$$

with

$$v = \frac{k^2 e^{ikr}}{4\pi r} e^{ik\frac{r}{2}(\frac{D}{2r})^2} \tag{14}$$

The total electric field for longitudinal and transverse configurations is given by Supplementary Equations (15) and (16), respectively.

$$\boldsymbol{E}^{\mathrm{ff,P}} \approx \boldsymbol{E}_{\mathrm{p1}}^{\mathrm{ff,P}} + \boldsymbol{E}_{\mathrm{p2}}^{\mathrm{ff,P}} + \boldsymbol{E}_{\mathrm{m1}}^{\mathrm{ff,P}} + \boldsymbol{E}_{\mathrm{m2}}^{\mathrm{ff,P}} \tag{15}$$

$$\boldsymbol{E}^{\mathrm{ff,S}} \approx \boldsymbol{E}_{\mathrm{p1}}^{\mathrm{ff,S}} + \boldsymbol{E}_{\mathrm{p2}}^{\mathrm{ff,S}} + \boldsymbol{E}_{\mathrm{m1}}^{\mathrm{ff,S}} + \boldsymbol{E}_{\mathrm{m2}}^{\mathrm{ff,S}} \tag{16}$$

By means of Supplementary Equations (17) and (18), the scattered intensity for longitudinal ($I^{\mathrm{P}}$) and transverse ($I^{\mathrm{S}}$) configurations can be obtained, taking into account the polarization of the receiving device

$$I^{\mathrm{P}} \approx r^2 \left| \boldsymbol{E}^{\mathrm{ff,P}} \cdot \boldsymbol{e}_{\mathrm{y}} \right|^2 \quad (17)$$

$$I^{\mathrm{S}} \approx r^2 \left| \boldsymbol{E}^{\mathrm{ff,S}} \cdot \boldsymbol{e}_{\mathrm{x}} \right|^2 \quad (18)$$

The coefficient $r^2$ is introduced in order to provide the normalized far-field intensity.

As it can be observed through Supplementary Equations (10-11) the scattered intensity in far-field in forward/backward for the longitudinal configuration is determined by the *y* component of the induced electric dipoles ($p_{1\mathrm{y}}$, $p_{2\mathrm{y}}$) and by the *x* component of the induced magnetic dipoles ($m_{1\mathrm{x}}$, $m_{2\mathrm{x}}$) in each one of the particles. $p_{1\mathrm{y}}$ (resp. $p_{2\mathrm{y}}$) is the result of the incident electric field and the field created by the *y*-component of the induced electric dipole in particle 2 (resp. particle1). $m_{1\mathrm{x}}$ (resp. $m_{2\mathrm{x}}$) is determined by the incident magnetic field and the field created by the *z*-component of the induced electric dipole and by the *x*-component of the induced magnetic dipole in particle 2 (resp. particle1).

For the transverse configuration, the scattered intensity in far-field in forward/backward direction (Supplementary Equations (12-13)) is given by the *x*-component of the induced electric dipoles ($p_{1\mathrm{x}}$, $p_{2\mathrm{x}}$) and by the *y*-component of the induced magnetic dipoles in each one of the particles ($m_{1\mathrm{y}}$, $m_{2\mathrm{y}}$). $p_{1\mathrm{x}}$ (resp. $p_{2\mathrm{x}}$) is the result of the incident electric field and the field created by the *x*-component of the induced electric dipole and by the *z*-component of the induced magnetic dipole in particle 2 (resp. particle1). $m_{1\mathrm{y}}$ (resp. $m_{2\mathrm{y}}$) is determined by the incident magnetic field and the field created by the *y*-component of the induced magnetic dipole in particle 2 (resp. particle 1).

In the two configurations, the coupling between the particles brings out an additional *z* component for the induced electric and magnetic dipoles[1,2]. Unfortunately, these *z* components do not appear in the forward/backward direction field expressions. Nevertheless, the coupling effects are still present in the far-field expressions of the scattered field, as the *x* and *y* components dipolar moments are affected by the particles

interactions (see the coupling terms in Supplementary Equations (2) and (4), i.e., the non-null off-diagonal terms in the matrix system).

The SDCs are the result of the interference effects in far-field between the electric field scattered by the electric and magnetic dipoles excited in both particles. For that reason, in order to analyze these conditions, we rewrite the scattered intensity in the following way:

$$\frac{I^{\mathrm{P}}}{r^2} \approx \left|\boldsymbol{E}^{\mathrm{ff,P}}_{\mathrm{p1+p2}} \cdot \boldsymbol{e}_{\mathrm{y}}\right|^2 + \left|\boldsymbol{E}^{\mathrm{ff,P}}_{\mathrm{m1+m2}} \cdot \boldsymbol{e}_{\mathrm{y}}\right|^2 + 2\,Re(\boldsymbol{E}^{\mathrm{ff,P}}_{\mathrm{p1+p2}} \cdot \boldsymbol{E}^{\mathrm{ff,P*}}_{\mathrm{m1+m2}}) \quad (19)$$

$$\frac{I^{\mathrm{S}}}{r^2} \approx \left|\boldsymbol{E}^{\mathrm{ff,S}}_{\mathrm{p1+p2}} \cdot \boldsymbol{e}_{\mathrm{x}}\right|^2 + \left|\boldsymbol{E}^{\mathrm{ff,S}}_{\mathrm{m1+m2}} \cdot \boldsymbol{e}_{\mathrm{x}}\right|^2 + 2\,Re(\boldsymbol{E}^{\mathrm{ff,S}}_{\mathrm{p1+p2}} \cdot \boldsymbol{E}^{\mathrm{ff,S*}}_{\mathrm{m1+m2}}) \quad (20)$$

It corresponds to a sum of the amplitude of the electric field created by the induced electric dipoles plus the amplitude of the electric field created by the induced magnetic dipoles and the interferential term between them (first, second and third terms of the right hand side of Supplementary Equations (19-20), respectively). *Re* refers to the real part of a complex number and * denotes the conjugate complex.

According to Supplementary Equation (19) (Supplementary Equation (20)), the SDCs for longitudinal (transverse) configuration are observed to those wavelengths where the Supplementary Equation (21) (Supplementary Equation (22)) is fulfilled.

$$\left|\boldsymbol{E}^{\mathrm{ff,P}}_{\mathrm{p1+p2}} \cdot \boldsymbol{e}_{\mathrm{y}}\right|^2 = \left|\boldsymbol{E}^{\mathrm{ff,P}}_{\mathrm{m1+m2}} \cdot \boldsymbol{e}_{\mathrm{y}}\right|^2 \quad (21)$$

$$\left|\boldsymbol{E}^{\mathrm{ff,S}}_{\mathrm{p1+p2}} \cdot \boldsymbol{e}_{\mathrm{x}}\right|^2 = \left|\boldsymbol{E}^{\mathrm{ff,S}}_{\mathrm{m1+m2}} \cdot \boldsymbol{e}_{\mathrm{x}}\right|^2 \quad (22)$$

or equivalently, the SDCs are observed for *P*-incident polarization (*S*-incident polarization) when Supplementary Equation (23) (Supplementary Equation (24)) is satisfied

$$\rho^{\mathrm{P}} = \frac{2\,Re(\boldsymbol{E}^{\mathrm{ff,P}}_{\mathrm{p1+p2}} \cdot \boldsymbol{E}^{\mathrm{ff,P*}}_{\mathrm{m1+m2}})}{\left|\boldsymbol{E}^{\mathrm{ff,P}}_{\mathrm{p1+p2}} \cdot \boldsymbol{e}_{\mathrm{y}}\right|^2 + \left|\boldsymbol{E}^{\mathrm{ff,P}}_{\mathrm{m1+m2}} \cdot \boldsymbol{e}_{\mathrm{y}}\right|^2} = \pm 1 \quad (23)$$

$$\rho^{\mathrm{S}} = \frac{2\,Re(\boldsymbol{E}^{\mathrm{ff,S}}_{\mathrm{p1+p2}} \cdot \boldsymbol{E}^{\mathrm{ff,S*}}_{\mathrm{m1+m2}})}{\left|\boldsymbol{E}^{\mathrm{ff,S}}_{\mathrm{p1+p2}} \cdot \boldsymbol{e}_{\mathrm{x}}\right|^2 + \left|\boldsymbol{E}^{\mathrm{ff,S}}_{\mathrm{m1+m2}} \cdot \boldsymbol{e}_{\mathrm{x}}\right|^2} = \pm 1 \quad (24)$$

If $\rho = 1$, we are in presence of maximal constructive interferences. Instead, if $\rho = -1$, the interferences are destructive. This coefficient varies with the polarization case

$$\rho^{\mathrm{P}}(180°) = -\rho^{\mathrm{P}}(0°) \; = -2\, \varepsilon_{\mathrm{h}}\varepsilon_0 Z\, Re \frac{\left(p_{1y}^{\mathrm{P}}+p_{2y}^{\mathrm{P}}\right)\left(m_{1x}^{\mathrm{P}}+m_{2x}^{\mathrm{P}}\right)^*}{\left|p_{1y}^{\mathrm{P}}+p_{2y}^{\mathrm{P}}\right|^2+(\varepsilon_{\mathrm{h}}\varepsilon_0 Z)^2\left|m_{1x}^{\mathrm{P}}+m_{2x}^{\mathrm{P}}\right|^2} \tag{25}$$

$$\rho^{\mathrm{S}}(180°) = -\rho^{\mathrm{S}}(0°) = 2\, \varepsilon_{\mathrm{h}}\varepsilon_0 Z\, Re \frac{\left(p_{1x}^{\mathrm{S}}+p_{2x}^{\mathrm{S}}\right)\left(m_{1y}^{\mathrm{S}}+m_{2y}^{\mathrm{S}}\right)^*}{\left|p_{1x}^{\mathrm{S}}+p_{2x}^{\mathrm{S}}\right|^2+(\varepsilon_{\mathrm{h}}\varepsilon_0 Z)^2\left|m_{1y}^{\mathrm{S}}+m_{2y}^{\mathrm{S}}\right|^2} \tag{26}$$

In Supplementary Fig. 1 we plot the $\rho$ coefficient for the analyzed dimer configurations, for longitudinal and transverse configurations and for two interaction parameters ($d_0 = 2$ and $d_0 = 1/3$). For comparison, we also show the case corresponding to an isolated particle. Although (in the spectral region where the dipolar approximation holds) it is possible to find two different size parameter values ($q$) where Supplementary Equations (21-24) are fulfilled, we focus the rest of our analysis on that range around $q = 0.85$, where the near Zero-Forward and its "rotated" version ("near Zero-Backward" condition) are achieved. The study has been carried out for the forward direction, as it is the direction where we want an increase in the scattered intensity.

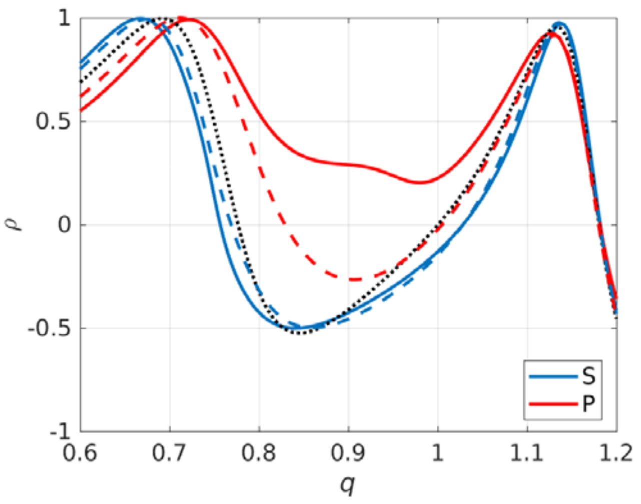


**Supplementary Figure 1**. Spectral behavior of $\rho$ coefficient. Interference coefficients $\rho^{\mathrm{P}}(180°)$ (red curve) and $\rho^{\mathrm{S}}(180°)$ (blue curve) for a HRID dimer, with different interaction parameters: $d_0 = 1/3$ (full line), $d_0 = 2$ (dashed line), and an isolated particle (black dotted line).

As it can be noted by means of Supplementary Fig. 1, when the interaction effects between the particles are weak, the obtained results are similar to those corresponding to an isolated particle, especially for the *S*-incident polarization configuration. The great similitude between the isolated particle and the dimer structure when $d_0 = 2$ comes from the fact that the terms $g_{yy}$, $g_{xx}$ and $g_{zx}$, which take into account the interaction between particles 1 and 2, are almost negligible. Indeed, $\lim_{D\to\infty} g(D) = 0.$ Therefore in the matrix

system detailed in Supplementary Equations (2) and (4), all the coupling terms disappear. In such a way, the induced electric and magnetic dipoles are mostly originated by the incident electric and magnetic fields, respectively.

However, for longitudinal configuration and strong interaction effects ($I^{\mathrm{P}}$, $d_0$ = 1/3), the spectral response is quite different to that of the isolated particle. In fact, it is observed that at the $q$ values where the near Zero-Backward condition is attained ($q$ around 0.85), the $\rho$ coefficient is never negative as in the rest of the analyzed configurations. In order to understand more deeply this behavior, in Supplementary Fig. 2 we show the spectral evolution of the two first terms of the right hand side of Supplementary Equations (19-20) for different distances between the particles of the dimer (gap). As it is observed, for $P$-incident polarization, as the distance between the particles (gap) decreases, both resonances (the one generated by the induced electric dipoles in both particles, $|\boldsymbol{E}_{\mathrm{p}}|^2$ and the one originated by the induced magnetic dipoles in both components of the dimer, $|\boldsymbol{E}_{\mathrm{m}}|^2$) are broadened. For the resonance originated by the induced electric dipoles, the broadening is due to the strong coupling between the induced electric dipoles in both particles. For the resonance generated by the induced magnetic dipoles, apart from the broadening, it is possible to observed two different contributions. The resonance at $q$ = 0.78 is due to the induced magnetic dipole in particle 1 (resp. 2) by the incident magnetic field and by the $x$ component of the induced magnetic dipole in particle 2 (resp. 1). The contribution observed at $q$ = 0.94 is originated by the induced magnetic dipole in particle 1 (resp. 2) by the $z$ component of the induced electric dipole in particle 2 (resp. 1). The electric dipole along the propagation direction of the incident radiation ($p_{2\mathrm{z}}/p_{1\mathrm{z}}$) is induced by the field of the primary magnetic dipole and it is not excited directly by the incoming wave. Due to the broadening, the condition expressed in Supplementary Equation (21) is not attained in the tails of the curves corresponding to $|\boldsymbol{E}_{\mathrm{p}}|^2$ and $|\boldsymbol{E}_{\mathrm{m}}|^2$ as it happens for weak interaction effects between the particles or for $S$-incident polarization. Instead of that, the directional effects are due to the interference between the sharp dipolar magnetic resonance with the tail of the broader dipolar electric mode, originating a Fano-like resonance. This behavior is responsible for the "near Zero-Backward" condition.

The results corresponding to the backward direction are opposite to those shown for the forward direction, due to the sign difference in the $\rho$ coefficient term. Indeed, as shown in Supplementary Equations (25-26), $\rho(180°) = -\rho(0°)$. Thus, constructive

interference in forward direction is associated to destructive interference in backward direction and vice-versa.

Through this analysis, it is clear that the interaction effects between both components of the dimer are responsible for the “near Zero-Backward” condition. As it has been previously reported in ref. 3, strong coupling is necessary to achieve a Fano resonance, (in our configuration, the dipolar electric mode is broadened due the interaction effects between the particles). However, the observation of the SDCs requires only the overlap of the independent electric and magnetic resonances, being less dependent on the interaction.

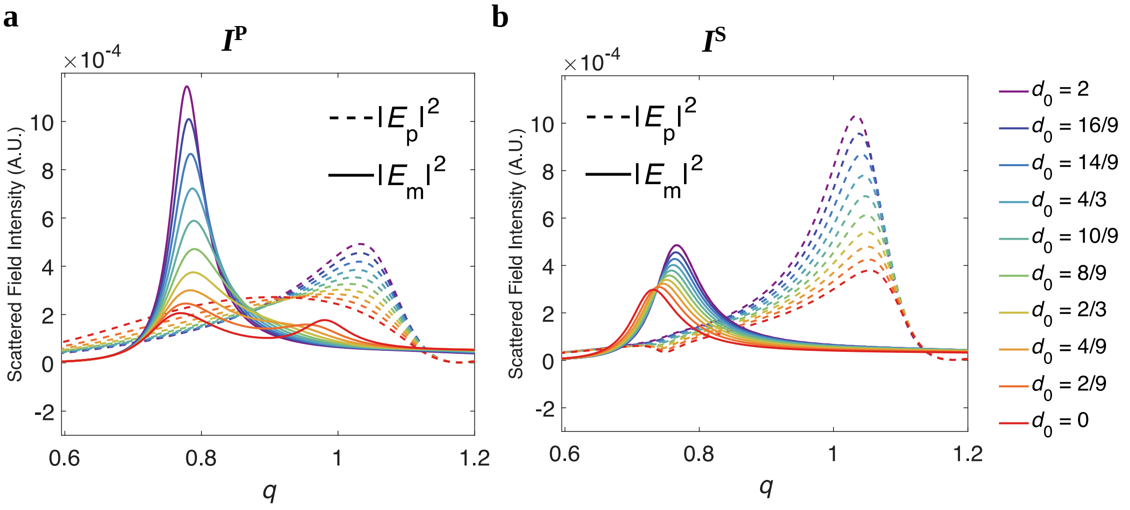


**Supplementary Figure 2**. Amplitude terms of Supplementary Equations (19-20). Electric field amplitude created by the induced magnetic (solid line) and electric (dashed line) dipoles in both particles of the dimer for different interaction parameters $d_0$ and polarizations of the incident radiation. **a)** Longitudinal configuration (*P*-incident polarization). **b)** Transverse configuration (*S*-incident polarization).

## Supplementary Note 2

**Scattering diagrams for a HRID isolated particle.** We have computed the angular distribution of the scattered intensity in far-field for a HRID isolated particle at the frequencies where the SDCs are observed for longitudinal and transverse configurations. In Supplementary Fig. 3, it is evidenced that at size parameter values around 0.85 (resp. 0.70) the incident radiation is mainly scattered in the backward (resp. forward) direction, being almost null (resp. null) in the forward (resp. backward) direction. The near Zero-Forward (resp. Zero-Backward) condition is attained.

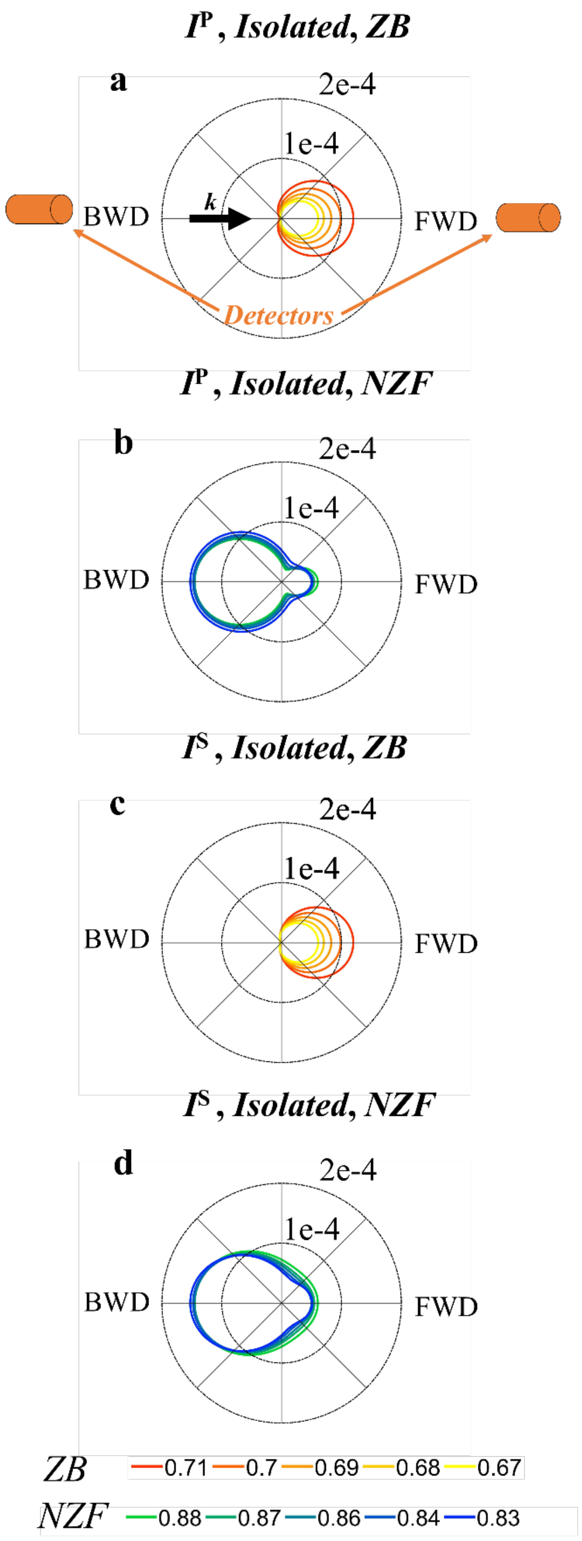


**Supplementary Figure 3.** Angular variations of scattered intensities. Scattering intensity diagrams in the scattering plane (*ZY*, see Fig. 1) at the frequencies where the SDCs are observed for an isolated HRID particle. In the first (**a**) and third (**c**) rows we show the diagrams corresponding to the Zero-Backward (*ZB*) condition ($q$ = 0.71, 0.70, 0.69, 0.68, 0.67) for longitudinal and transverse configurations, respectively. In the second (**b**) and fourth (**d**) rows we plot the scattering diagrams at the near Zero-Forward (*NZF*) condition ($q$ = 0.88, 0.87, 0.86, 0.84, 0.83) for *P*-incident polarization and *S*-incident

polarization, respectively. See bottom lines for color codes. The black arrow represents the direction of the incident radiation (***k***). The orange cylinders indicate the detectors positions. FWD stands for Forward direction and BWD stands for Backward direction.

## Supplementary references


1. Albella, P. *et al*. Low-Loss Electric and Magnetic Field-Enhanced Spectroscopy with Subwavelength Silicon Dimers. *J. Phys. Chem. C* **117**, 13573−13584 (2013).
2. Barreda, A. I. *et al*. Electromagnetic polarization-controlled perfect switching effect with high-refractive-index dimers and the beam-splitter configuration. *Nat. Commun.* **8**, 13910 (2017).
3. Shibanuma, T., Albella, P. & Maier, S. A. Unidirectional light scattering with high efficiency at optical frequencies based on low-loss dielectric nanoantennas. *Nanoscale* **8**, 14184–14192 (2016).
